# Researching the mental health needs of hard-to-reach groups: managing multiple sources of evidence

**DOI:** 10.1186/1472-6963-9-226

**Published:** 2009-12-10

**Authors:** Christopher Dowrick, Linda Gask, Suzanne Edwards, Saadia Aseem, Peter Bower, Heather Burroughs, Amy Catlin, Carolyn Chew-Graham, Pam Clarke, Mark Gabbay, Simon Gowers, Derek Hibbert, Marija Kovandzic, Jonathan Lamb, Karina Lovell, Anne Rogers, Mari Lloyd-Williams, Waquas Waheed, the AMP Group

**Affiliations:** 1 School of Population, Community and Behavioural Sciences, Whelan Building, University of Liverpool, Liverpool L69 3GB, UK; 2 Primary Care Research Group, 5th Floor, Williamson Building, University of Manchester, Oxford Road, Manchester, M13 9PL, UK; 3 School of Nursing, Midwifery and Social Work, University Place, University of Manchester Oxford Road, Manchester, M13 9PL, UK

## Abstract

**Background:**

Common mental health problems impose substantial challenges to patients, carers, and health care systems. A range of interventions have demonstrable efficacy in improving the lives of people experiencing such problems. However many people are disadvantaged, either because they are unable to access primary care, or because access does not lead to adequate help. New methods are needed to understand the problems of access and generate solutions. In this paper we describe our methodological approach to managing multiple and diverse sources of evidence, within a research programme to increase equity of access to high quality mental health services in primary care.

**Methods:**

We began with a scoping review to identify the range and extent of relevant published material, and establish key concepts related to access. We then devised a strategy to collect - in parallel - evidence from six separate sources: a systematic review of published quantitative data on access-related studies; a meta-synthesis of published qualitative data on patient perspectives; dialogues with local stakeholders; a review of grey literature from statutory and voluntary service providers; secondary analysis of patient transcripts from previous qualitative studies; and primary data from interviews with service users and carers.

We synthesised the findings from these diverse sources, made judgements on key emerging issues in relation to needs and services, and proposed a range of potential interventions. These proposals were debated and refined using iterative electronic and focus group consultation procedures involving international experts, local stakeholders and service users.

**Conclusions:**

Our methods break new ground by generating and synthesising multiple sources of evidence, connecting scientific understanding with the perspectives of users, in order to develop innovative ways to meet the mental health needs of under-served groups.

## Background

### The scale of the problem

According to the World Health Organization, half of all people with ill health in Western Europe have mental illness, with the majority coming into the diagnostic categories of anxiety and depression [1]. Mental health problems impose substantial emotional, social and economic burdens on those who experience them, their families and carers, and society as a whole [2-4]. A wide range of clinical interventions [5], collaborative care [6], self-management [7], and social and community initiatives [8] are effective in improving the lives of people experiencing common but disabling mental health problems such as depression and anxiety. However many people with high levels of mental distress are disadvantaged, either because care is not available to them in the right place and time, or because when they do access care their interaction with care-givers deters help-seeking or diverts it into forms that do not address their needs [9].

Groups with inadequate access to primary care include people from black and minority ethnic (BME) communities, asylum seekers, homeless people and adolescents with eating disorders [10-13]. Groups who receive inadequate help when they do access primary care include elders, people with advanced cancers, those at risk of long term sickness absence and people with medically unexplained symptoms (MUS) [14-17].

The extent of commonality of issues across these hard-to-reach groups means a combined approach is likely to be most effective. The Social Exclusion Unit's report on mental health confirms that people from these groups face considerable barriers to getting their mental health needs addressed [3]. Women from BME communities, homeless people, asylum seekers, and elderly people living alone, for example, often experience severe and persistent social difficulties. Engagement and communication are inherently problematic in the case of adolescents with eating disorders [18] and women from ethnic minorities [19]. We consider that lessons learnt here may have wider implications for other groups of people whose mental health problems are managed within primary care.

### The need for a new methodological approach

Existing methodological approaches go some way towards enabling a thorough understanding of the mental health needs of such hard-to-reach groups, and to generating new solutions to those needs: but none is sufficient.

Quantitative evidence from randomised trials, and subsequent systematic review and meta-analysis, provide valuable information about what works and for whom, but is of little help in explaining why. Qualitative sources of evidence are needed to find out why current practices may not work, and what might help to improve them. Published qualitative sources may not directly address key questions, such as problems with access: hence there may be a need to turn to detailed interview transcripts, or to ask actual and potential service users and providers directly about specific issues. Grey literature, produced by service providers or advocacy groups, is a useful source of evidence for the current priorities of policy makers and service providers. Stakeholders must be involved not only in answering research questions, but also in conceptualising the questions to be asked. It is then essential to find effective and valid ways of synthesising evidence in order to generate and test potential solutions.

Other groups have proposed ways of combining some of these multiple sources of evidence, to inform the design of complex interventions. Dixon-Woods et al [20] propose a valuable range of methods for synthesising quantitative and qualitative evidence, but their focus is primarily on published research. The perspectives of service users and health professionals have been combined with systematic literature review to guide study design by Robinson et al [21] and Richards et al [22]. Al-Janabi et al have combined economic evaluation with meta-ethnography and participant interviews [23]. Lovell et al [24] have extend this with a modelling procedure which includes both synthesis of diverse sources of published research evidence and a consensus process to guide the delivery of an intervention. However in these studies the involvement of stakeholders, especially service users, tended to be limited both in numbers and in scope, with relatively little enquiry into views on problem formulation or presentation. None of these approaches have reported reviewing grey literature on existing services.

In the context of the mental health needs of hard-to-reach groups, where there is uncertainty not only about how services should be configured but also about how mental health needs should best be understood, we therefore considered it necessary to undertake a more rigorous and comprehensive approach to evidence generation and synthesis.

### Aims and objectives

The aim of our research and development programme (AMP) is to increase equity of access to high quality primary care mental health services for hard-to-reach groups [25]. The objective of this paper is to describe how we tackled the methodological challenges inherent in clarifying the mental health needs of people from these groups; in identifying relevant evidence-based primary care services, and barriers and facilitators for access to them; and then in developing a portfolio of credible and acceptable interventions.

## Methods

### Underlying perspectives

We began with the assumption that members of hard-to-reach groups are not passive victims suffering mental health problems, but are people who interpret and respond to experiences, and are capable of mounting challenges to external forces bearing upon them [26]. We considered the interrelationships between macro-level societal and institutional factors in the creation of mental health problems amongst hard-to-reach groups, and the interventions offered to them within or through primary care. We focused on key clinical outcomes, exploring how to adapt service configurations to meet patients' needs rather than changing patient presentation to fit in with existing services. We put patients' experiences and expressed needs at the centre of care, and addressed the broader practice and policy contexts in which these are located.

We adopted a whole system approach to identify needs, barriers and facilitators to developing a range of credible and acceptable interventions. This meant that we could - and should - examine diverse sources of evidence [27].

**Figure 1:**
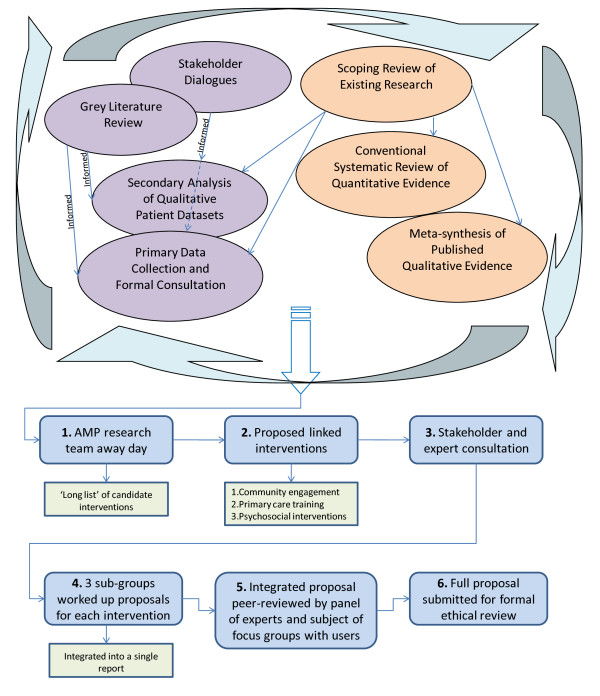
**Methods of evidence generation and synthesis**.

The approach we have taken is presented schematically in Figure 1, to which the rest of this section of the paper refers. We carried out the literature reviews and stakeholder exercises in parallel, rather than in series, in order to give stakeholders the opportunity to inform the literature reviews.

### Identifying key concepts

We began with a scoping review to develop a map of key concepts concerning access to primary care, and to identify the range of current interventions that have been used to improve access to care. This review was generic and not restricted to mental health: the intention was to capture the full range of relevant concepts and definitions. Initially, members of the research team were asked to identify key papers and books relating to access. This database was augmented by a search of electronic databases using a range of terms relating to access, combined with a search filter developed at the National Primary Care Research and Development Centre in Manchester to identify conceptual and theoretical literature (the scoping review strategy is available on request from the authors). Candidate interventions around access were developed, starting with the list of interventions developed by the EPOC group of the Cochrane Collaboration [28], and refined through focussed literature searches and reflection on the developing conceptual map.

**Figure 2:**
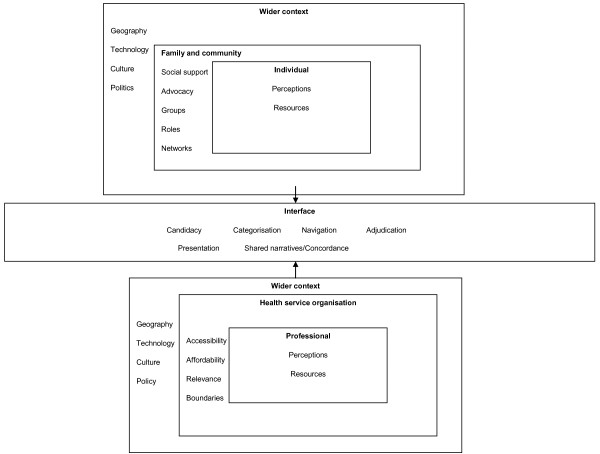
**Key concepts in access to health care**.

The major components of our scoping review are presented in Figure 2.

We draw attention to three key concepts emerging from this review.

*Recursivity: *refers to the ways in which illness behaviour is both enabled and constrained by the interactions that take place between individuals and health professionals in health service settings [29]. The ability of health professionals to communicate effectively with patients may reinforce or discourage health action in the future [30].

*Candidacy: *describes how people's eligibility for healthcare is determined between themselves and health services [31]. Candidacy arises from ongoing negotiation, influenced by a wide number of factors. Health services are constantly defining and redefining the legitimate objects of health services. In response, patients are also trying to make sense of this process.

*Cultural competence:* mental health services have traditionally not been responsive to ethnic and cultural minorities [32]. One criticism of the notion of 'cultural competence' is that it often focuses on particular BME groups and ascribes characteristics to individuals based on a crude group membership [33]. An alternative conception is that cultural competence requires clinicians to take into account the individual values, beliefs and practices of the patient (which may or may not reflect their membership of a group). In this way, cultural competence can be seen as a specific form of patient-centredness [34], where the clinician 'tries to enter the patient's world, to see the illness through the patient's eyes' [35].

### Gathering evidence

We used these concepts to underpin the direction of our investigation of the mental health needs of people from under-served groups, and how best to meet them, drawing evidence from six separate sources.

#### Systematic review

We systematically reviewed published evidence concerning the effectiveness of candidate interventions in improving access to primary care for patients with mental health problems. The population under review was patients with mental health problems in primary care. This included diagnosed disorders and non-specific problem categories such as 'psychosocial problems' and 'stress'. We included the range of candidate interventions that have been used to improve access to care identified in the scoping review. Comparisons included no treatment, usual care, and other candidate access interventions. We expected that most published quantitative evaluations of the effectiveness of interventions would focus on effectiveness and cost-effectiveness outcomes, and that benefits of access would be implied rather than stated. The focus was on identifying the amount of evidence for each group, the range of interventions that had been trialled, and the broad pattern of the results.

The review began with a search of the Cochrane Database of Systematic Reviews and DARE for previous reviews of candidate access interventions. This was followed by a search of MEDLINE, EMBASE, CINAHL and PsychINFO using a range of terms relating to candidate access interventions, combined with an RCT and primary care filter. The full text of abstracts identified by the search were obtained and eligibility judged by two reviewers. Papers where eligibility was difficult to judge were assessed by other members of the research team and disagreements resolved by discussion. Eligible papers (n = 133) had data extracted onto standardised pro-forma and quality was assessed using standard criteria. Results were presented as narrative or analysed using conventional meta-analytic techniques where appropriate.

#### Meta-synthesis of published qualitative literature

By identifying commonalities across disparate published qualitative sources, meta-synthesis works in a similar way to a meta-analysis of disparate published quantitative sources, in generating a more powerful level of evidence in relation to a particular issue. This qualitative review examined the experiences, and attitudes to mental health treatment of identified hard-to-reach groups in their social contexts, in order to shed light on participants' understandings of the processes of access and their experiences of the health system.

Search terms generated from known papers and prior research were combined with a previously developed qualitative research filter, tested and adapted to run across MEDLINE, CINAHL, EMBASE, PsychINFO, ASSIA and WoK. The resulting abstracts were assessed for relevance and a sub-set of papers was then extracted. The British Sociological Association's quality criteria [36] were used to generate a definitive set of 21 papers for the final synthesis. Key findings were extracted to standardised pro-forma and were synthesized within and across groups using the classic lines-of-argument approach proposed by Noblit and Hare [37].

#### Dialogues with stakeholders

This was a two stage process. We formed a stakeholder steering group through actively establishing relationships with stakeholders. This group then helped us to identify the mental health needs of our exemplar hard-to-reach groups and the extent to which primary care currently meets these needs; and the barriers and facilitators to, and critical components of, high quality mental health services in primary care.

Potential stakeholders were identified using snowballing techniques [38]. Dialogues were conducted with individuals and groups, either by telephone or face-to-face. We arranged 53 dialogues in individual or group format with 83 key opinion leaders and informants from relevant organisations, including health professionals, clinical academics, other service providers, commissioners, and service user and carer representatives of our exemplar groups. Stakeholders were also invited to suggest relevant grey literature, inform the systematic reviews, and suggest potential contacts for subsequent interviews with former, current and potential service users.

Audio-recordings or notes of dialogues were made, with the consent of the participants. A dialogue analysis template was completed for each encounter. Each stakeholder was sent a draft copy of their dialogue analysis for any amendments and additions. Each completed document was then analysed using a thematic analysis template. Each analyst identified over-arching and common themes within exemplar groups, and noted synergies and differences between groups. Finally, we produced a parallel thematic analysis which portrayed the emerging themes identified by each analyst, and developed a synthesis of findings with recommended interventions and approaches to improving access.

#### Grey Literature

The aim of the grey literature review was to find out what mental health services, of relevance to primary care, were currently available, planned or potentially accessible for members of hard-to-reach groups, primarily across the north-west of England.

Using the wide range of specialist expertise available in the research team, we sourced relevant published material. We did not seek a comprehensive collection, rather we sought to identify and address gaps in the peer reviewed literature. Documents (n = 120) gathered in this pragmatic search were were mainly statutory health sector or voluntary sector reports. Individual summaries were prepared for each document, with a focus on recommendations relevant to the eight exemplar groups and their access to mental health services. We also looked for any guidance on the design of interventions to improve access, and examples of good practice and innovation.

#### Secondary analysis of qualitative datasets

The aim of this study was to gather evidence from service users about key issues in relation to access to primary care mental health services, arising from the scoping review. This analysis was complementary to the meta-synthesis in that it allowed access to the full text of interview transcripts, rather than the illustrative material presented in published papers.

Qualitative studies contributing data for secondary analysis were sampled by convenience from those already collected in other projects within the wider research activity of the team. Research team members selected particular datasets from those available according to the team's judgment on their relevance to our research objectives. As the original studies were designed to answer various research questions related to common mental health problems and mental well-being, transcripts that were judged by the primary researcher as irrelevant were discarded after discussion. The next step was a random selection of transcripts from each of seven datasets, generating 33 transcripts for analysis.

The process of collaborative secondary analysis was based on the methods described by May *et al *[39]. Initial analysis using new conceptual perspectives, naïve to original research findings was conducted in parallel with re-analysis conducted by primary researchers or by comments from primary researchers on initial findings. The next step was cumulative comparative analysis, where initial findings could be complemented by additional theoretical sampling, and final summaries of findings for each study were sent to primary researchers for validation. Finally a condition comparative analysis considered similarities and differences between findings for each study group.

#### Interviews with (potential) service users

The aim of this study was to continue the process of gathering evidence from service users and carers about key issues in relation to access, focussing on members of hard-to-access groups for whom adequate evidence was not available from the meta-synthesis of published qualitative literature, or from the analysis of existing qualitative datasets.

This study therefore sought interviews with service users and carers from five BME communities (South Asian, Irish, Chinese, Somali and Polish), and with asylum seekers, homeless people and adolescents with eating disorders. Recruitment started with flyers displayed at locations across Liverpool and Manchester, where members of our target groups would be likely to attend. The interviews were conducted at locations convenient to the participants: usually this was in office space provided by the recruiting organisation, but on occasion in the participant's own home. Interpreting was facilitated by M-Four Translations, at Manchester City Council.

Basic demographic data was collected for each participant. The interviews (n = 34) were semi-structured, with a topic guide developed with reference to the scoping review and interviews with stakeholders (see above). All the interviews were audio-recorded and transcribed verbatim.

Transcripts were analysed on a case-by-case basis, focusing on: the ways in which the participants understood their emotional health and well being; their attitudes towards help-seeking for emotional distress; and their experiences if they had tried to access mental health services. A comparative case analysis within the exemplary groups identified group specific themes. A further comparative case analysis across the complete dataset identified over-arching themes. Ethical approval for this aspect of the programme was given by Wrightington, Wigan and Leigh Research Ethics Committee [reference 08/H1014/39].

### Synthesising evidence and generating solutions

We synthesised the evidence gained from these multiple and diverse sources, using a consensus process [40], to inform our development of candidate interventions which would take account of known barriers while remaining sensitive to the needs, preferences and priorities of our exemplar hard-to-access groups and other stakeholders. We focused on mental health-related services which have the potential to be commissioned by primary care trusts, including those delivered by non-statutory organisations.

Our procedures for generating the candidate interventions involve a series of interactions between the research team and local stakeholders, actual and potential service users from our exemplar groups, and a panel of national and international academic experts in the field of primary care mental health (see Figure 1).

1. We began this process with a tightly structured away day for the entire AMP research team, at which we considered the totality of evidence generated from the various sources indicated above, and used facilitated small-group techniques to generate an initial 'long list' of candidate interventions.

2. We then produced a working document, as a basis for consulting with the wider health care community. This document proposed a set of linked interventions in three domains:

• enhanced community engagement, to raise awareness of the potential benefits of interaction with primary care;

• increased sensitivity of primary care teams to the mental health needs of people from hard-to-reach groups;

• the design and implementation of a range of sensitised psycho-social interventions.

3. Members of our stakeholder group and our panel of experts were invited to comment on the working document, using electronic pro-formata.

4. The research team then formed into three sub-groups, to work up detailed proposals for each of the proposed domains, taking account of service user, stakeholder and expert comments. We then integrated the working groups' reports into a single document.

5. We invited our panel of experts to subject the detailed integrated proposal to a formal peer-review process; and, in parallel, held a series of consultations with service users and primary care teams to gather views on the utility and acceptability of these proposals.

6. Finally, we submitted the full proposal for formal ethical review through the NHS IRAS system.

### Ilustration of methods

To illustrate how these processes worked, we describe how we focused our intervention strategy on specific hard-to-reach groups - older people and people from BME communities, who are experiencing depression and medically unexplained symptoms - and why we chose particular sorts of interventions.

The direction of inquiry was informed by the concepts emerging from the scoping review, principally recursivity, candidacy and cultural competence. Our starting point was to target groups who are least likely to access primary care (such as BME communities) and those who receive substandard care if they do obtain access (such as older people). Our systematic review of psychosocial interventions identified the largest number of positive outcome studies in these two groups. Findings from our meta-synthesis and our secondary analysis highlighted differing ways in which mental health problems may be expressed, with a strong emphasis on physical manifestations of suffering: hence we realised the importance of including physical symptoms. And we received convergent advice from our local stakeholders, our panel of experts and our participating primary care trusts that older people and BME groups should be given highest priority.

The rationale for our choice of the form and content of psychosocial interventions to be tested is derived from a synthesis of our multiple sources of evidence, including our programme team meetings. This is summarised in Table 1.

**Table 1:** Evidence for content of psycho-social interventions relating to older people and BME communities

*Source of Evidence*	How to make the intervention acceptable	How to make the intervention accessible	Evidence base	Who should be involved in the delivery	Service considerations
**Systematic review**		People can benefit from existing interventions	Consistent evidence that older people could benefit from various psychological treatments; and evidence that modified psychological treatments may be effective for people from BME groups.		
**Grey literature**	Focus on the individual not the condition Culturally appropriate Interventions should work to normalise mental health and recalibrate the boundaries between mental health, physical health and social life	Reaching out to the community Collaborative working Users need information on how to access services		Advocacy to help people navigate their way through the service	Low intensity and social support Services should be bottom up
**Stakeholder perspective**	Communicative, flexible, holistic, integral, positive, proactive, responsive				
**Secondary analysis of qualitative data**	Pluralistic, adaptive, holistic, resonant and socially conscious Somatisation of mental suffering Cultural sensitivity Use of metaphors and individuals explanatory models	Stigma prevents access and help seeking			Social deprivation and isolation Improving availability and reach-ability; understanding and improving experience and expectation of care
**Service-user perspectives**	Isolation and loneliness Decreasing stigma	Lack of knowledge about available services			Lack of compassion and communication from health professionals
**Qualitative review-Meta synthesis**	Reengagement with the wider social world Use of diagnostic labels may be counter productive Build on current strengths	Information to make informed choices		Respect and interest in culture	Willingness by health professionals to understand service users views of themselves
**Conclusions drawn on Synthesis day**	Working with patients' explanatory models Focus on both psychological and social issues	Signposting to relevant services Culturally acceptable Variable site delivery Multi delivery system	Evidence-based psychological interventions	Community 'champions' Understanding of local services and referral criteria Peer-led and professional-led	

We can see from this table how evidence from stakeholders, service users and qualitative literature all emphasised the need for health care organizations and health professionals to promote anti-discriminatory attitudes and behaviours. Reviewing the different sources of evidence about increasing access to interventions, we found that the content of psychosocial interventions needs to be presented in ways which are culturally acceptable, incorporate physical difficulties and decrease social isolation. It also became clear, from the meta-synthesis, the secondary analysis and the stakeholder perspectives, that there is a need to incorporate service users' own explanatory models, language and metaphors within the psychosocial interventions. Stakeholder and service user perspectives indicated that the interventions should be available in a number of delivery modes, and in both healthcare and community settings.

The trustworthiness of our approach can be further assessed by the extent to which emergent findings challenged the prior assumptions of the research team, and by the ways in which we addressed divergent evidence.

With regard to our prior assumptions, we have been presented with three major challenges by our evidence synthesis: the first concerns the complexity of defining access, which we are now considering further; the second is the unexpected extent of existing information on effective psychosocial interventions for BME groups and older people, which enables us to refine our planned interventions to a greater level of specificity than anticipated; and the third is the strong emphasis on the physical embodiment of suffering, which will influence the content of our educational interventions with primary care teams.

Evidence from different information sources was more likely to provide differing emphases, rather than formally to diverge. For example, in Table 1 we see that service users tended to emphasise the problem of isolation and loneliness, whereas the qualitative metasynthesis focused more on potential solutions inherent in re-engagement with the wider social world. Amongst service users, there was divergence of views about acceptable care for their mental health problems, with preferences ranging from specialist psychiatric care, through general practice to complementary approaches such as acupuncture or aromatherapy. We overcame this discordance by incorporating the concept of medical pluralism, expressed in the statement that culturally competent care needs to address individual values, beliefs and practices and 'see the illness through the patient's eyes' [34].

## Discussion

The AMP programme is designed to increase equity of access to high quality mental health services in primary care. The first steps to achieving this are to find out what high quality services exist, and what the barriers and facilitators are to their successful implementation for people from under-served groups. In this paper we have explained how and why we gathered and then synthesised evidence from multiple sources, in order to understand the problems and generate potential solutions.

### Strengths and limitations

The major strength of our methodological approach is that it has enabled us to answer a set of important questions which could not adequately be addressed by simpler or more conventional means. No single source of evidence would have provided adequate information, or had sufficient credibility, to allow confident understanding of the problems being presented or their potential solutions. Conversely, each provided a unique facet of the overall picture.

The systematic review could tell us what psychosocial interventions worked for which groups of people, but not how or why they worked nor, importantly, how such interventions might be better accessed. The grey literature review and stakeholder interviews could tell us what services are available or planned, but not what potential service users thought about them. The meta-synthesis and secondary analysis could tell us about actual and potential service users' views on the formulation of mental health problems and on the services that might meet their needs, but were restricted by the issues they primarily sought to address. The interviews with actual and potential service users then enabled us to fill gaps in knowledge. Finally, our processes of evidence synthesis and refinement, involving several iterations between the AMP team, stakeholders, service users and external experts, were focused towards the development of an acceptable and credible intervention strategy. We consider our comprehensive methodological approach to be particularly useful in circumstances such as this, when substantial uncertainty exists regarding not just the range of possible solutions to a given health problem, but also regarding the nature and presentation of the health problem itself.

There are also indirect advantages to the extensive methods we used, for example the enhancement of skills within the research team, and developing links with local services and stakeholders.

Such a complex approach to evidence gathering and synthesis does have its limitations. First, our aim to examine the needs of a wide range of hard-to-access groups, within and amongst which there is considerable diversity of demands and expectations, has an inherent tendency to generate solutions which are generic rather than specific. Second, it was not possible to for all the objectives of all our evidence- gathering strands to be fully realised: our grey literature review, for example, was limited by the willingness of service providers to respond to our requests; and our accesss to interviews with Polish service users was more limited than anticipated. Third, we are aware of the complexities of gathering information from stakeholders, who may act as brokers and filters of information from their communities, effectively censoring information they deem inappropriate to share with outsiders [41]. Fourth, there remains the risk that, despite all the external advice and review procedures built into this process, the perspectives and prejudices of the AMP team may have exerted undue influence on the conclusions and consequent outcomes.

### Implications

We believe that our comprehensive approach to evidence gathering and synthesis is unique, and could be seen as a gold standard method for informing the design of new complex interventions. While other research groups have successfuly synthesised quantitative and qualitative sources of research evidence, and incorporated consensus procedures to identify key interventions [20-24], we are not aware of previous examples of research teams including stakeholder and service-user perspectives within their development stage in the integrated, synergistic manner achieved here. We commend our methodological approach to others planning innovative, research-based complex interventions in health care, particularly where both the health problem under consideration and the possible solutions to it are subject to heterogeneity of understanding and interpretation.

We will separately publish papers reporting the main findings from this process of evidence generation and synthesis. We will use the results of this process to test interventions which have relevance to our exemplar groups. This intervention phase will be based on the three previously mentioned linked domains of community engagement, primary care quality and sensitised psychosocial interventions. All chosen interventions will be subjected to rigorous evaluation. We will deploy a range of suitable methodologies, including case-control, before-and-after and qualitative designs, to examine uptake, acceptability, satisfaction, knowledge and understanding on the part of both providers and users of services. Finally, we will move from experimentation to implementation, and test new strategies to enable the successful dissemination into routine practice [42,43] of those interventions which have demonstrable acceptability and efficacy.

As with the MRC's guidance on developing and evaluating complex interventions [44] questions must remain about the ultimate value of such an exhaustive approach to evidence gathering and synthesis. It will not be clear after a single application whether all this work was worthwhile, in terms of both time and money, or whether it might have been possible to do it much more efficiently. The test will be the quality of the insights generated from the research programme, and the acceptability and effectiveness of the interventions developed within the programme.

## Conclusions

We already have a good understanding of what works in primary care mental health for the general population. The AMP programme has developed a set of methods which link that knowledge with what is known about barriers to access for under-served groups, integrating scientific understanding with the perspectives of stakeholders and service users and carers, in order to propose creative new ways to meet the specific needs of members of under-served groups.

Our programme brings together previously separate streams of research and development activity. In so doing it reduces duplication of effort and enables synergies. Lessons can thus more readily be learned about effective research and clinical methods and their dissemination into routine practice. In addition to the intrinsic value of our programme's content to the priorities and needs of the UK's National Health Service, it may serve as a model for co-operation between health service and academic institutions in the successful prosecution of primary care research and development activities, and as a basis on which universities may engage ethically and effectively with their local communities [45].

## Abbreviations

AMP: Improving Access to Mental Health in Primary Care; ASSIA: Applied Social Science Index and Abstracts; BME: black and minority ethnic; CINAHL: Cumulative Index to Nursing and Allied Health Literature; DARE: Database of Abstracts of Reviews of Effects; EMBASE: Exerpta Medica Database; EPOC: Effective Practice and Organisation of Care; IRAS: Integrated Research Application System; MUS: medically unexplained symptoms; RCT: randomised controlled trial; WoK: Web of Knowledge.

## Competing interests

The authors declare that they have no competing interests.

## Authors' contributions

CD and LG conceived the study, and designed the research in collaboration with all the other authors. PB conducted the scoping review with HB, and the systematic review with SA. JL and MK contributed to refinement of the scoping review and its translation into research methods of subsequent phases. JL, LG and AR undertook the meta-synthesis. CCG, PC, SE, DH and MK conducted and analysed the stakeholder and service user contributions. DH and AC conducted the grey literature review. MK and CCG led the secondary data analysis. WW, MK, JL, DH and KL led the intervention planning sub-groups. CD drafted the manuscript. All authors read and approved the final manuscript.

The AMP group is composed of people who supported the research but did not contribute directly to this paper: Dawn Edge, Margaret Goddard, Christina Ireland, Nicky Lidbetter, Rosalind McNally, Rajan Madhok, Gabrielle Marr, Sarah Peters, Joanne Reeve and Katherine Rowley

## Pre-publication history

The pre-publication history for this paper can be accessed here:

http://www.biomedcentral.com/1472-6963/9/226/prepub
